# pH-sensitive Self-associations of the N-terminal Domain of NBCe1-A Suggest a Compact Conformation under Acidic Intracellular Conditions

**DOI:** 10.2174/092986612802762642

**Published:** 2012-10

**Authors:** Harindarpal S Gill

**Affiliations:** Case Western Reserve University, Department of Physiology & Biophysics, 10900 Euclid Avenue, Robbins Building E549, Cleveland, OH 44106-4970

**Keywords:** NBCe1, SLC4, bicarbonate, cotransporter, light scattering, surface plasmon resonance.

## Abstract

NBCe1-A is an integral membrane protein that cotransports Na^+^ and HCO_3_^-^ ions across the basolateral membrane of the proximal tubule. It is essential for maintaining a homeostatic balance of cellular and blood pH. In X-ray diffraction studies, we reported that the cytoplasmic, N-terminal domain of NBCe1-A (NtNBCe1-A) is a dimer. Here, biophysical measurements show that the dimer is in a concentration-dependent dynamic equilibrium among three additional states in solution that are characterized by its hydrodynamic properties, molar masses, emission spectra, binding properties, and stabilities as a function of pH. Under physiological conditions, dimers are in equilibrium with monomers that are pronounced at low concentration and clusters of molecular masses up to 3-5 times that of a dimer that are pronounced at high concentration. The equilibrium can be influenced so that individual dimers predominate in a taut conformation by lowering the pH. Conversely, dimers begin to relax and disassociate into an increasing population of monomers by elevating the pH. A mechanistic diagram for the inter-conversion of these states is given. The self-associations are further supported by surface plasmon resonance (SPR-Biacore) techniques that illustrate NtNBCe1-A molecules transiently bind with one another. Bicarbonate and bicarbonate-analog bisulfite appear to enhance dimerization and induce a small amount of tetramers. A model is proposed, where the Nt responds to pH or bicarbonate fluctuations inside the cell and plays a role in self-association of entire NBCe1-A molecules in the membrane.

## INTRODUCTION

Bicarbonate, also known as baking soda, plays an important metabolic role in acid-base balances. Solute carrier family 4 (SLC4) transporters are integral membrane proteins that transport HCO_3_^**–**^ ions across epithelial cells throughout the body [1-6]. The SLC4 family consists of five functionally distinct groups [7, 8]. Each group contains many splice variants that differ in their cytoplasmic domains [9]. The sodium bicarbonate cotransporter, NBCe1-A, is one major variant of the electrophoretic group, which without ATP transports Na^+ ^and HCO_3_^**–**^ ions out of the cell at a ratio that yields a net negative charge across the membrane. In the case where NBCe1-A is located at the basolateral membrane of the proximal tubule, NBCe1-A reabsorbs 80% of the filtered HCO_3_^– ^from the lumen to blood, thereby playing a major role in regulating blood pH [8]. Similar to other human SLC4s, NBCe1-A contains a large (~400 aa) cytoplasmic, N-terminal domain (NtNBCe1-A), of which little is known about its role in transport or regulation [9-11]. However, defects in NtNBCe1-A result in severe autosomal recessive disorders that are associated with acidic blood pH, vision loss, dental abnormalities, and mental retardation [12-18]. 

The function of the cytoplasmic domains of the entire human SLC4 family is under investigation [11, 19, 20]. Ae1, a Cl/HCO3 cotransporter also known as *Band 3*, is another SLC4 family member and plays a structural role in the integrity of the membrane of the red blood cell (RBC). Early work on the cytoplasmic, N-terminal domain of Ae1 (NtAe1) describes NtAe1 as an anchoring site for the membrane skeleton of the RBC and several peripheral proteins [21, 22]. These binding partners include ankyrin, protein 4.1, hemoglobin, and other cytoplasmic proteins. NtAe1 also has been described to exist in equilibrium among three conformations referred to as “a conformational equilibrium” that are reversible and pH-dependent [23]. These conformations are characterized by an alkaline-induced increase of 11 Å in Stokes radius, a two-fold increase in intrinsic fluorescence that displays a biphasic or sigmoidal curve as a function of pH, an increase in protein segmental mobility, and a loss of ankyrin affinity, without any major change in protein secondary structure [19, 24]. A hypothetical model, based on the NtAe1 crystal structure, was suggested to explain the radial increase at elevated pH, where a domain movement in each monomer expands the length of the dimer [25]. Alternative studies on the hydrodynamic properties of full-length Ae1 purified from RBC suggest that Ae1 is in a pH-dependent equilibrium among monomer, dimer, and tetrameric states [26]. NtAe1 is responsible for driving these self-associations of full-length Ae1; the membrane-spanning region by itself does not demonstrate a pH dependence of association [27]. 

Similar protein-binding partner and biophysical studies as those above for NtAe1 have yet to be performed for NtNBCe1-A, although there has been indirect evidence by mutational analysis that a substrate tunnel exists within NtNBCe1-A [11]. Biochemical studies have been hampered by the lack of stability of NtNBCe1-A in solution. We previously created specific steps, such as the streptomycin precipitation in low salt buffers, which eliminated fractions of low solubility early in the purification to reduce the precipitation [9]. The studies here retrospectively illuminate the conditions of NtNBCe1-A necessary to keep it completely mono-dispersed and soluble for an indefinite period of time. For the first time, *in vitro* functional assays for NtNBCe1-A are presented and should better define the role of the Nt among all SLC4 family members. This leads to a hypothetical model of a SLC4 cotransporter that is placed in the membrane—with bicarbonate ions modulating the self-association of Nt molecules.

## MATERIALS AND METHODS

### Purification and Protein Concentration Measurements of NtNBCe1-A

The purification of NtNBCe1-A (residues 1 to 362) followed the protocol as detailed in Gill *et al*. [9], except for the following adjustments: i) the wash buffer for the nickel-affinity step was adjusted to pH 7.0 instead of pH 8.0; that is, the wash buffer contained 50mM HEPES pH 7.0, 200mM NaCl, 20mM Imidazole pH 7.0, 0.2%(v/v) 2-mercapto-ethanol; ii) NtNBCe1-A molecules were retained on the nickel resin overnight, thereby eliminating the subsequent need for the ammonium-sulfate precipitation step that previously was necessary for stability and storage; iii) the elution buffer contained 50mM HEPES 7.0, 200mM NaCl, 300 mM Imidazole pH 7.0, 0.2%(v/v) 2-mercaptoethanol; and iv) the eluent (~10 ml) was then directly applied without concentration over a Highload Superdex-200pg (26/60) column (GE Healthcare, Piscataway, NJ) equilibrated with 50mM Tris pH 7.0, 150mM NaCl, 1mM TCEP. Protein concentration was determined by measuring the absorbance at 280 nm implemented in the Beer-Lambert Law using an extinction coefficient of ~730 mL/(g*cm). The extinction coefficient was measured by applying the elution fraction from the nickel column over a Superdex-200 HR (10/30) column (GE Healthcare) connected online to a refractive-index detector (Optilab, Wyatt Technologies, Santa Barbara, CA). Stokes radii were calculated by measuring peak elution volumes and comparing to protein standards (Biorad #151-1901, Hercules, CA) consisting of bovine thyroglobin 670 kDa, bovine γ-globin 158 kDa, chicken ovalalbumin 44 kDa, horse myoglobin 17 kDa, vitamin B12 1.35 kDa.

### Size-exclusion Chromatography with MultiAngle Light Scattering (MALS-SEC)

**Figure 1. The hydrodynamic radius of NtNBCe1-A as a function of pH:**
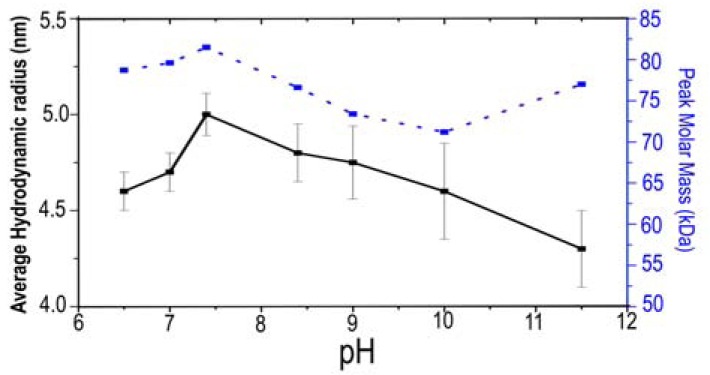
The average hydrodynamic radius (${\bar{r}}_{H}$) of NtNBCe1-A molecules exhibit an inverted V-shaped curve over the pH range 6.5 to 11.5 as measured by dynamic light scattering DLS (solid curve). The polydispersity (or standard deviation) of the apparent hydrodynamic radii (R_H_) are relatively low at slightly acidic pH values (Pd < 10%) and relative high at alkaline values (Pd > 15%). Note that a hot spot or increase of R_H_ occurs at pH 7.4 and that the RH values are larger at acidic pH compared to those at basic pH. Measurements by DLS also were compared to measurements by size-exclusion chromatography SEC (dashed curve), which yielded a similar trend in radii corresponding to molar masses estimated from peak elution volumes.

For Fig. **1**, from the Superdex-200 (26/60) column, samples (0.5 ml) of ~2 mg/ml NtNBCe1-A in 50mM Tris 7.0, 150mM NaCl, and 1mM TCEP were applied over a Superdex-200 HR (10/30) column (GE Healthcare) connected serially online with a three-angle light-scattering detector (MiniDAWN TREOS, Wyatt Technologies) and a refractive index detector (Optilab, Wyatt Technologies), thus allowing for out-of-equilibrium molar-mass measurements. The column was repeatedly equilibrated with buffers containing 50mM Tris, 150mM NaCl, 1mM TCEP for each pH analyzed. Data was processed using the ASTRA V software package (Wyatt Technologies). A WTC-030S5 column (Wyatt Technologies) was alternatively used in MALS-SEC measurements for comparison. For Fig. **4B**, samples (0.5 ml) at ~10 mg/ml were used on the same Superdex-200 HR (10/30) column.

### Dynamic Light Scattering (DLS)

From the Superdex-200 (26/60) column, the peak fraction after purification also was analyzed in batch mode (50 µl) using a dynamic-light scattering detector (DYNA PRO, Wyatt Technologies) that is supplemented with a single static detector. Here, the peak fraction already contained ~2.5 mg/ml protein. Concentration was avoided to minimize dimer self-associations for hydrodynamic (R_H_) measurements. The purification was repeated, equilibrating the Superdex-200 (26/60) column sequentially with buffers containing 50mM Tris, 150mM, 1mM TCEP for each pH used for R_H_ measurements. Alternatively, after passing NtNBCe1-A over the same column with buffer containing 50mM Tris pH 6.5, 150 mM NaCl, and 1mM TCEP, the entire fractions were pooled and concentrated to 2.5 mg/ml using a MW cut-off filter of 30 kDA (Millipore). Samples (100 µl) were then dialyzed in micro-dialysis cups (Millipore) against 100 ml of buffers for each pH. Hydrodynamic data collection again was performed in batch mode. Finally, for in-equilibrium molar-mass measurements at pH 7.4, the peak fraction contained 1.1 mg/ml. 

### Steady-state Fluorescence

Samples (0.5 ml) of ~2 mg/ml NtNBCe1-A in 50mM Tris 7.0, 150mM NaCl, and 1mM TCEP were applied over a Superdex-200 HR (10/30) column (GE Healthcare). The column was repeatedly equilibrated with buffers containing 50mM Tris, 150mM NaCl, 1mM TCEP for each pH used in measurements. Peak fractions for each run were diluted to 70 µg/ml in a volume of 200 µl and analyzed using a fluorescence spectrophotometer (FluoroLog^®^-3, Horiba Scientific, Edison New Jersey, NJ). The Raman-scattering peak was subtracted from spectral data. Alternatively, the samples after initial purification were diluted to 70 µg/ml in a volume of a 100 µl in buffers containing 50mM Tris, 150mM NaCl, 1mM TCEP for each pH measurement and analyzed with a M1000 plate reader (Tecan, Durham, NC). 

### Circular Dichroism (CD) Spectroscopy

Far-UV CD spectra of 2-3 μM NtNBCe1-A in TRIS-buffered saline at varying pH values and at 25 °C were recorded on an Aviv 202A CD spectrometer (Aviv Biomedical). CD spectra were measured between 200 to 250 nm and background corrected.

### Surface Plasmon Resonance

The surfaces of two flow cells (FC1 and FC2) of a carboxymethylated-dextran (CM-5) chip were washed with 50 mM NaOH in parallel using a flow rate of 10 μl/min for 3 min using a Biacore T-100 (GE Healthcare). NtNBCe1-A was immobilized on the surface of FC2 via amine coupling. Two types of experiments were performed: firstly, for simple confirmation of self-association, a resonance-unit (RU) signal of 12,000 was achieved with NtNBCe1-A (ligand) concentration of 50 μg/ml in 10 mM acetate buffer pH 5.5 at flow rate of 10 μl/ml. The chip then was blocked with 1M ethanolamine (pH 8.5) at a flow rate of 10 μl/ml for 7 min. FC1 served as a reference cell following mock-immobilization with buffer alone. Serial-diluted solutions of NtNBCe1-A (analyte) in 10 mM Hepes pH 7.4, 150 mM NaCl, 2 mM EDTA, 0.005% surfactant P20 (HBS-P) were passed over both flow-cells. Binding traces were recorded for at least five concentrations of analyte. Each binding cycle was performed at room temperature with a constant flow rate of 30 μl/min for 8 min. Each regeneration cycle was performed with HBS-P buffer following spontaneous dissociation. Secondly, for kinetic rate constants determination, NtNBCe1-A was immobilized onto a new chip yielding a RU of 500. Using similar conditions as above, curves obtained after subtraction of the reference and the buffer signals were fitted using the BIAevaluation (GE Healthcare). For bicarbonate-binding experiments, fresh solutions of bicarbonate were made and adjusted to pH 7.5. Aliquots were placed into vials sealed with rubber caps, which were punctured with needles to withdraw solution. Experiments were carried out within a few hours. Experiments also were similarly performed with bisulfite, which is a stable bicarbonate analog that unlike bicarbonate does not require the presence of CO_2_. 

## RESULTS

### Hydrodynamic Radius Measurements of NtNBCe1-A are pH Sensitive

Fig. **1** shows a plot of hydrodynamic radius (or Stokes radius) **R_H_** as a function of pH. Measurements using dynamic-light scattering DLS (Fig. **1**, solid curve) indicate that the observed **R_H_** average (${\bar{r}}_{H}$) of 4.6 nm at slightly acidic pH is in close agreement with theoretical calculations [28] of 4.2 nm for a molecular mass expected of pure dimers. The observed increase in ${\bar{r}}_{H}$ to 5.0 nm at neutral pH is consistent with a small amount of dimer-dimer interactions, which would have a theoretical value of 5.6 nm for a molecular mass expected of pure tetramers, a very large conformational change within the Nt, or both. Thereafter, the observed **R_H_** values begin to drop as pH is moderately elevated. This drop appears consistent with an increasingly amount of monomer in a monomer-dimer mixture. The NtNBCe1-A samples are monodispersed at acidic and neutral pH values, both with a polydispersity (Pd) < 10% or 13% respectively. The samples are polydispersed (Pd ~ 15 to 25%) at moderately alkali pH and above. As shown in Fig. **1** (dashed curve)**,** peak molar-mass measurements using size-exclusion chromatography are in agreement with **R_H_** measurements of DLS. Peak-elution volumes correspond to peak-elution times that ideally are proportional to **R_H_**. For NtNBCe1-A, molecules generally shift toward higher volumes corresponding to a decrease in **R_H_**, as the pH of the column-equilibration buffer is gradually increased. Note in Fig. **1** that the change in the rate of diffusion through the column media mimics the directly observed increase or jump in **R_H_** at neutral pH measured by DLS. 

### Molar-mass Measurements Reveal a Dynamic Equilibrium Among Three States

**Figure 2. Molar mass of NtNBCe1-A as a function of pH.**
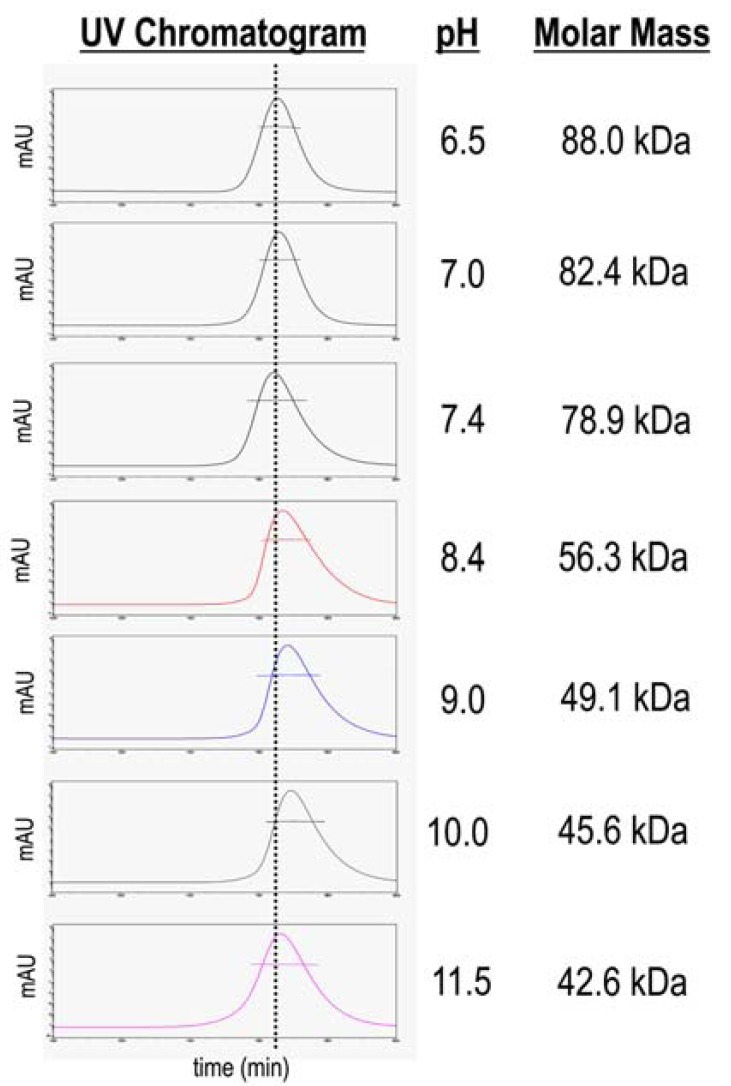
The elution profiles of NtNBCe1-A are shown at varying pH with its observed average molar-mass values as measured by MALS-SEC. The molar mass trend demonstrates equilibrium among monomers and dimers. Dimer disassociation or an increase in monomer formation is observed as pH increases (significantly above pH 8). Note at pH 11.5, the molar mass is consistent with a solution of monomers. The fact that the molar mass trend over the entire pH range differs from the trend in R_H_ measurements suggests a conformational or state change is occurring. The molar mass bars are represented on a logarithmic scale. Further, the dotted line helps to illustrate that the peak elution volume (or Stokes radius) shifts as a function of pH that were plotted in Fig. **1**, dashed curve. These shifts are due to the change in the relative amounts of monomer and dimer. Note that at pH 11.5, the peak is extremely broad in comparison to the peak at pH 6.5, which has a similar peak elution volume, suggesting non-specific interactions that would explain the only discrepancy between SEC and DLS measurements of R_H_.

Out-of-equilibrium (online) and equilibrium (batch) molar mass measurements demonstrate NtNBCe1-A is in a dynamic equilibrium among three states. In online experiments, measurements by ‘multiangle-light scattering–size exclusion chromatography’ (MALS-SEC) demonstrate a dynamic equilibrium among three molecular masses that correspond to monomer, dimer, and dimer-dimer interaction. Fig. **2** illustrates the molar mass of NtNBCe1-A applied to a gel-filtration column as a function of pH. At neutral pH, NtNBCe1-A appears to be in equilibrium among all three states. The average molar mass varies in the range of 78.9–82.4 kDa, which is in close agreement with the theoretical value of 81.2 kDa that corresponds to that of a dimer. As with the DLS experiments above, each individual fraction of monomer and dimer species can be pronounced by moderate acidic and alkaline changes in pH. At acidic pH, the UV trace appears to be most symmetrical. The uniformity is consistent with the DLS data above that suggested NtNBCe1-A is most monodispersed at acidic pH. As pH is gradually increased further, the molar masses gradually decreases and the tail to the right of each peak is increasingly pronounced. The decrease of molar mass and tail reflect an increase in the amount of monomer in a monomer-dimer mixture, yielding average molar mass values that are an average of monomer and dimer species. At pH 11.5, the observed molar mass of 42.6 kDa is in agreement with the theoretical value of 40.6 kDa corresponding that of a monomer, suggesting that the monomer now predominates the solution. 

In batch experiments, measurements using a single light-scattering detector also demonstrate that the NtNBCe1-A is in equilibrium. As the concentration of NtNBCe1-A is decreased, the molar mass decreases. The average molar mass at pH 7.4 is observed to be 82 kDa at 1.1 mg/ml, 52 kDa at 0.2 mg/ml, and 43 kDa at 0.02 mg/ml. The decrease in molar mass is indicative of a dynamic equilibrium. Fluctuations within each concentration were observed and may reflect self-associations that are analyzed further below. 

### Tryptophan Fluorescence and CD Spectra Indicate Conformational Changes that are pH Dependent

**Figure 3. Tryptophan emission spectra as a function of pH:**
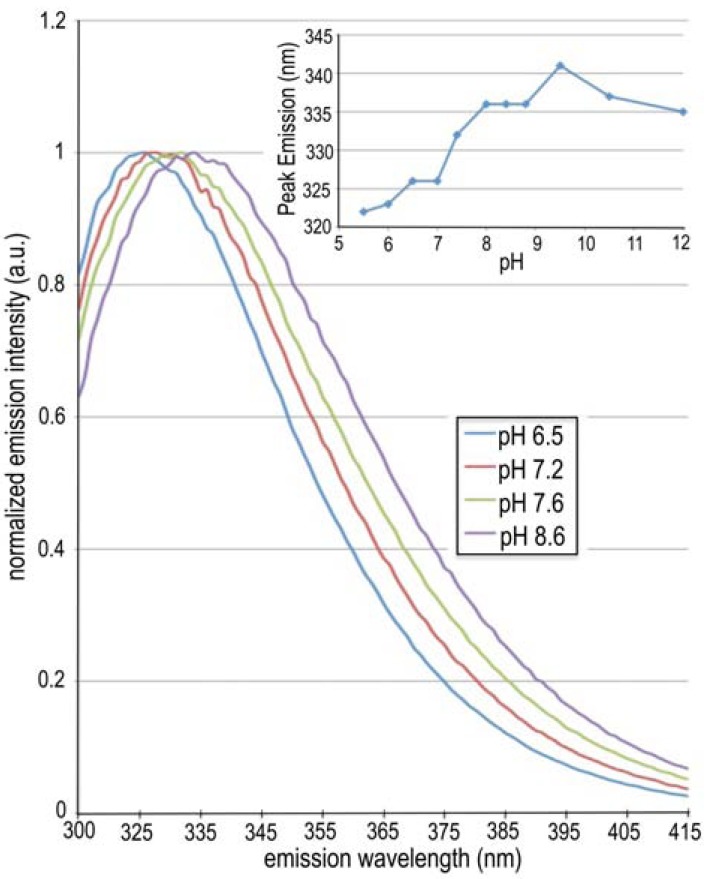
The emission spectra of NtNBCe1-A exhibit an incremental Stokes shift as pH is gradually raised from 5.5 to 11.5. The figure shows pH values indicated in the legend increasing from left to right in the spectra. An 11 nm Stokes shift in total is observed. Note that the shift is continuous within the physiological pH range. The continuous transition here partially reflects subtle local conformational changes. The shift above pH 8 is largely associated with an increasing population of dimer dissociation into monomers, thereby exposing W341 to solvent that would normally be buried at the dimer interface. The spectra were collected with a traditional fluorometer, which are in agreement with the inset readings, which were sampled with a sensitive fluorescence plate reader.

Fig. **3** shows the tryptophan-fluorescence spectral curve of NtNBCe1-A at varying pH. The spectra exhibit continuous Stokes shifts in peak emission. At acidic pH, the NBCe1-A dimer exhibits a peak emission at 326 nm. At neutral pH, NBCe1-A molecules exhibit a Stokes shift of 6 nm toward longer wavelength. Likewise, further increases in pH result in NtNBCe1-A molecules to gradually emit light at longer wavelengths. At extreme alkaline conditions (pHThe emission spectra of NtNBCe1-A exhibit an incremental Stokes shift as pH is gradually raised from 5.5 to 11.5. The figure shows pH values indicated in the legend increasing from left to right in the spectra. An 11 nm Stokes shift in total is observed. Note that the shift is continuous within the physiological pH range. The continuous transition here partially reflects subtle local conformational changes. The shift above pH 8 is largely associated with an increasing population of dimer dissociation into monomers, thereby exposing W341 to solvent that would normally be buried at the dimer interface. The spectra were collected with a traditional fluorometer, which are in agreement with the inset readings, which were sampled with a sensitive fluorescence plate reader.

11.5), where monomers predominate, the peak emission occurs at 336 nm. To compare, NtNBCe1-A also was unfolded in the presence of 6 M guanidine-chloride. Unfolded NtNBCe1-A emits light at 346 nm, typical to that of other unfolded proteins [29, 30], supporting that the smaller observed peak emission changes by pH are due to conformational and/or state changes rather than unfolding of the molecule. CD spectra support the robust nature of NtNBCe1-A despite extreme changes in pH. CD spectra and mean residue ellipticities were obtained for the entire spectrum of pH values each yielding minima at 208 and 220 nm, suggesting similar structures. The fact that there is negative ellipticity at all pH values, except when 6M guanidine hydrochloride is present, means that the secondary structure of NtNBCe1-A is indeed intact. This is consistent with Fig. **2**, which shows that NtNBCe1-A still elutes in the linear separation volume of the gel-filtration column and thus molecules are folded despite large pH changes. Lastly, the peak emission shifts are fully reversible if pH is lowered from pH values < 8 and largely reversible if lowered from pH values > 8. This reversibility also indicates that the tertiary fold is retained and that two distinct state changes occur above and below pH 8.

### Self-associations of NtNBCe1-A

**Figure 4. Self-association of NBCe1-A:**
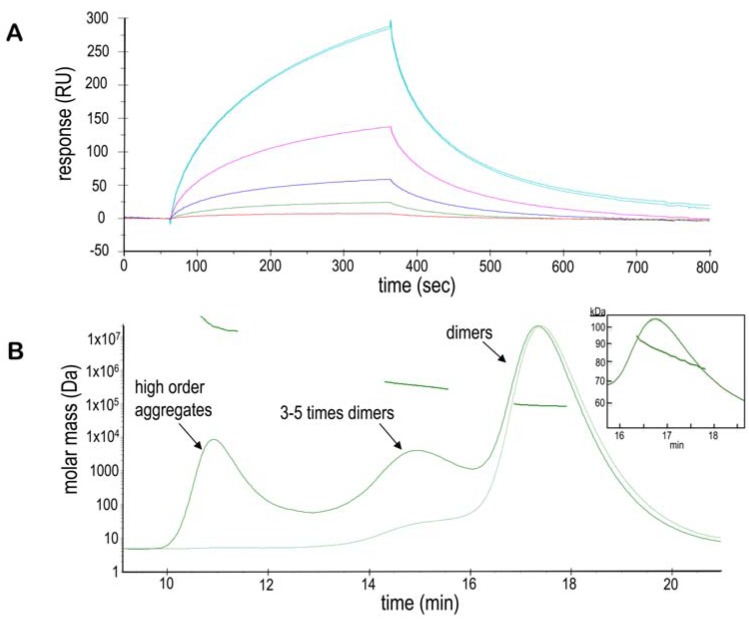
**A**. A blank-substracted sensogram of an SPR-Biacore experiment demonstrates NtNBCe1-A self-associate and self-dissociate. Here, NtNBCe1-A as free analyte is flown over the same as immobilized ligand. Each curve from top to bottom corresponds to a different concentration of analyte: 400 µg/ml, 80 µg/ml, 1.6 µg/ml, 0.32 µg/ml, 0.064 µg/ml with a RU of 500 of protein immobilized on the surface. The shape of the curves demonstrates slow-on and fast-off rates, whose kinetics are complicated by a dynamic equilibrium of states. Although there maybe a minor amount nonspecific clustering at 400 µg/ml, overall the binding appears to be specific and reversible, negating the need to regenerate the immobilized-NtNBCe1-A surface between concentration cycles. As a control, free NtNBCe1-A molecules were flowed over a blank CM5 sensor chip that showed virtually no significant binding to the reference chip. The concentration series are reference subtracted and blank subtracted. **B**. The self-association of the NtNBCe1-A dimer is further illustrated by a MALS-SEC experiment at pH 7.6, where higher-order oligomers or aggregates are increasingly observed as noted by the bars above each peak. Measurements show that these interactions can yield prominent, self-associated species upto 3-5 times of the mass of a dimer. The upper thick curve is the light scattering, and the lower thin curve is the UV trace. Finally, the monomer-dimer equilibrium can be easily noted in the inset by the fact that the molar mass trend across the far right peak slopes downward, where it is ~93 kDa to the left of the peak corresponding to a small amount of dimerdimer interaction and ~74 kDa at the right shoulder of the peak reflecting a mixture of monomer and dimer.

Measurements by surface plasmon resonance (SPR-Biacore) directly confirm self-association interactions, which are significantly observed at neutral pH only. NtNBCe1-A (ligand) was immobilized on a chip while the same solution of NtNBCe1-A (analyte) was flowed over it. Fig. **4A** shows the label-free binding of NtNBCe1-A at varying concentrations onto the NtNBCe1-A immobilized chip. NtNBCe1-A molecules can be seen to transiently or reversibly bind to the chip, yielding typical curves that exponentially increase for association and decrease for disassociation. Because the chip briefly was treated with blocking agent at pH 8.5 after immobilization of compact NtNBCe1-A dimers (See METHODS AND MATERIALS), Fig. **1**-**3** suggest a small amount of the dimers disassociated on the chip, and the observed self-associations of analyte maybe due to monomer self-association and/or dimer self-associations. Whether we are looking at a monomer self-association (monomer-dimer equilibrium) or dimer self-association (dimer-tetramer equilibrium) depends on the concentration. Low concentration favors monomer-dimer equilibrium. High concentration of the Nt dimer results in high-order self-associations, as evident in Fig. **4B** and in Gill & Boron (2006) (Fig. **2b**). Isolated tetramers and 3-5x dimer fractions were reapplied onto a SEC column resulting in a single-dimer peak again as those in Fig. **2**, showing that the clusters are reversible*.*

### Bicarbonate Binding

**Figure 5. Bicarbonate binding.**
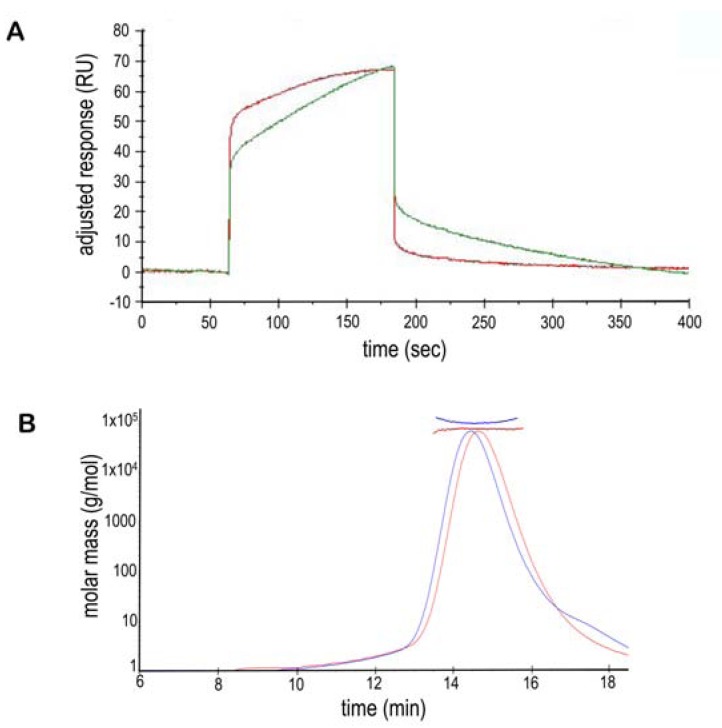
**A**. NtNBCe1-A self-disassociation is shown with and without the presence of bicarbonate. The upper curve that falls to ~0 RU in 180 sec is with 0 mM bicarbonate and the lower curve that falls to ~0 RU in 400 sec is with 10 mM bicarbonate. Note that 10 mM bicarbonate slows disassociation, or rather stabilizes NtNBCe1-A self-association, compared to 0 mM bicarbonate or those depicted in Fig. **4A**. The presence of bisulfite yielded similar results. **B**. The binding of NtNBCe1-A by bicarbonate analog–bisulfite–is demonstrated by a MALS-SEC experiment. The right-shifted curve is NtNBCe1-A with 0 mM bisulfite, and the left-shifted is NtNBCe1-A with 10 mM bisulfite. Note by the bars above the peaks that molar mass increases from ~79 to ~105 kDa in the presence of bisulfite, suggesting an increase in the amount of dimer self-association. Also note that the red and blue chromatogram traces differ in their shape. The left-shifted, revealing a clear shoulder peak, suggests discrete species or a decrease in the monomer population in the presence of bisulfite.

Employing the sensitivity of the SPR-Biacore, bicarbonate ions can be observed to bind NtNBCe1-A at low millimolar concentration (~2 mM) yielding a RU of 8. This value is in agreement with theoretical or R_max_ values of 9 with an R_L_ value of 9100, which corresponds to the RU of immobilized protein. As shown in Fig. **5A**, when bicarbonate is present in the solution during NtNBCe1-A self-association experiments, bicarbonate helps to stabilize the self-association interaction. The disassociation rate of the self-association interaction is three to four times slower (600 sec) as compared to those experiments with NtNBCe1-A (180 sec) by itself in the analyte solution. This binding enhancement is also true when bisulfite—a stable bicarbonate analog—is present in the analyte solution, yielding similar results. MALS-SEC indicates that bisulfite binds NtNBCe1-A, enhancing dimerization and high-order self-associations on the chip. Fig. **5B** demonstrates that the average molar mass increases ~25 kDa at neutral pH compared to those values listed above, the monomer-dimer equilibrium is disturbed as apparent by the shoulder at the peak tail where monomers normally predominate, and a minor (~2-fold) increase of high-order self-associations is observed in the MALS trace.

## DISCUSSION

### Monomer - Dimer - High Order Self-association Equilibrium

**Figure 6. Mechanism of self-associations:**
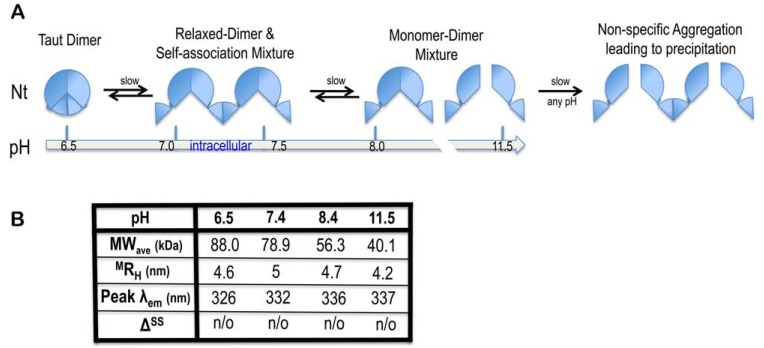
A schematic diagram of NtNBCe1-A in equilibrium (**A.**) is correlated to a summary of its the biophysical properties (**B.**). MW_ave_ is the average molecular weight, ^M^R_H_ is the measured hydrodynamic radius, Peak λ_em_ is the peak emission wavelength, Δ^SS^ is the change in secondary structure, n/o means not observed. The differences in trend between MW_ave_ and ^M^R_H_ and the shift in Peak λ_em_ suggest that conformational and/or state changes are occurring.

Based on the aforementioned data, the observed dynamic equilibrium of NtNBCe1-A under physiological conditions can be de-convoluted into three distinct states as illustrated in Fig. **6A**. In a first state, individual dimers exist in a taut conformation. In a second state, a conformational change occurs that allows dimers to relax and transiently bind one another or self-associate. In a third state, individual dimers are relaxed to the point where they disassociate into monomers. Intracellular bicarbonate levels or pH fluctuations can influence the relative amounts of individual states in the equilibrium. Fig. **6B** gives a summary of the biophysical properties of each state that corresponds to the schematic representation in Fig. **6A**.

### Comparison of Hydrodynamic Radii Profiles between NtAe1 and NtNBCe1-A

Although the **R_H_** of NtAe1 and NtNBCe1-A are both sensitive to pH changes, they appear to display different hydrodynamic properties. In the case of NtAe1, as pH increases from low pH, the **R_H_** of NtAe1 apparently strictly increases yielding a sigmoidal-shaped curve. At extreme alkaline pH, the **R_H_** of NtAe1 is at its maximum with a value of ~6.7 nm [19]. In the case of NtNBCe1-A, as pH increases from low pH, the **R_H_** temporarily increases at neutral pH and then slowly decreases thereafter yielding an inverted V-shaped curve. Notably, **R_H_** measurements at pH 11.5 are lower than those for compact dimers at pH ≤ 6.5. This smaller value at extreme alkaline pH is expected for a solution of nearly pure monomers. The unexpected difference in trend of **R_H_** curves could simply reflect a difference in protein sequences or rather affinity of the monomers in the dimer complex. However, at least in the case of NtNBCe1-A, there is no evidence for a global-conformational change at alkaline pH that extends the radius as described for NtAe1 [25]. Because the MALS-SEC technique is independent of shape changes, the gradual drop of molar mass at elevated pH is in agreement with an increase in the monomer population of the monomer-dimer equilibrium. There is rather evidence for 'local' conformational changes in NtNBCe1-A that have yet to be characterized for NtAe1. These subtle changes are detected here at neutral pH by **R_H_**, fluorescence, and SPR measurements. On the other hand, artifacts of elevated pH could mislead to a sigmoidal-shaped curve such as for NtAe1. For example, if NtNBCe1-A had been briefly exposed to elevated pH and then concentrated, the measurements for **R_H_** do become uniformly elevated at all pH. These elevated **R_H_** measurements reflect non-specific aggregation as revealed by MALS-SEC measurements. Here, the Nt slowly becomes unstable, a property that is addressed below. 

### Stability in Solution at High-concentrations (> 2 mg/ml)

As previously described in Gill *et al*. [9], a challenge to the storage and application of NtNBCe1 is that it undergoes a *slow*, time-dependent precipitation at high concentrations, such as ~10–15 mg/ml as typically used in crystallization trials, thereby periodically changing the concentration of the protein solution until it reaches concentrations less than ~2 mg/ml. This happens between 0-22°C incrementally after ~2-5 hrs. Generally, the protein solution holds up better at room temperature. Freezing it at -20°C results in the immediate precipitation of ~70-90% of the protein content upon thawing. Buffer stabilizers such as glycerol, varied pH conditions in the final-exchanged or gel-filtration buffer, and salt variations do not prevent the slow precipitation. However, as described here in METHODS AND MATERIALS, careful attention to keep the pH of each buffer 7.0 or less from the beginning of the purification enabled long-term stability. Values of pH > 8 shift the equilibrium of NtNBCe1-A toward monomers. Monomers then subsequently bind non-specifically to other monomers when highly concentrated. This can be considered as a fourth state in Fig. **6A**. These interactions irreversibly self-associate into insoluble complexes that fall out of solution in random waves over hours to days—even if pH is immediately reversed to neutral pH. That is, monomers are not stable at high concentrations for an extended period of time. Conversely, NtNBC1-A molecules that have only been exposed to acidic or neutral pH are stable for weeks. Although no precipitation is observed here, after two weeks at room temperature or 4°C, half of the dimers are dissociated into monomers as judged by MALS-SEC.

### Intracellular pH Sensitivity by NBCe1-A

It is unclear whether NtNBCe1-A directly responds to slight changes in intracellular pH or bicarbonate levels via deprotonation of key residues and/or to binding partners that modulate or shield charges on the Nt that induce conformational changes similar to those observed here. With that said, there are no binding partners known for NtNBCe1-A. Although trafficking chaperons and other integral proteins are likely needed to bind NBCe1-A, peripheral cytosolic proteins that regulate transport or increase stability analogous to those that bind NtAe1 in the RBC remain to be elucidated if they exist.

### Intrinsic Fluorescence Changes

There are four tryptophans that can be grouped into two environments. W341 is located at the monomer-monomer interface, and the W81, W87, W101 are clustered and partially buried within the molecule, as judged by homology modeling in X-ray diffraction studies with NtAe1 [10]. Thus, the longer wavelength (red shift) at alkaline pH in emission spectra can be accounted for by the disassociation of dimer into monomers, which would directly expose W341 to solvent, based on crystal models. However, local conformational changes at neutral pH also can be explained by changes in the environments of the three remaining tryptophan near the far cytosolic side of the Nt. 

### Model of a SLC4 

**Figure 7. Cartoon of a SLC4 member in the membrane:**
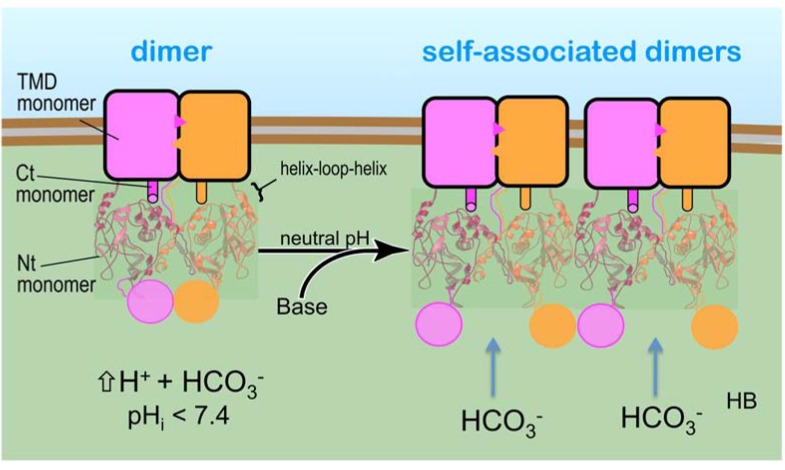
The figure shows the Nt to be isolated in a closed state (left) and self-associated in an open state under normal physiological conditions (right) in the cell. The self-association of dimers is governed by local conformational changes that are induced by intracellular pH or bicarbonate levels. The SLC4 is modeled using the crystal model of NtAe1 [25] and the low resolution, homology model derived from X-ray diffraction studies of NtNBCe1-A [10]. NtAe1 from the RBC shares a 37% identity in amino acid sequence to NtNBCe1-A. The high identity extends to all SLC4 family members and reveals that all Nt domains share the same structural fold. Each monomer of the dimer extends an arm, which is inserted into the other monomer. The two arms are interlocked, symmetrically holding the dimers together. This interaction implies that the transmembrane domain of NBCe1-A, shown by the adjacent rectangles, necessarily must also form symmetrical dimers. The studies for NtAe1 [27] and here for NtNBCe1-A suggest that the Nt drives self-association of NBCe1-A dimers in the membrane.

The functional roles of SLC4 family members can be divided into their Nt and transmembrane domains (TMD). The Nt of a SLC4 does the work of sensing intracellular pH or bicarbonate levels, promoting self-associations, and possibly acting as a selection filter via internal substrate conduits. The TMD of a SLC4 provides a scaffold for substrate transport across the membrane and conceivably regulates transport by occlusions that define the cotransporter class of integral membrane proteins. Structurally, as illustrated in Fig. **7**, the Nt hangs like a gondola via flexible loops from the TMD. The Nt dimer is placed next the membrane with its N-termini end facing far away from the membrane. The helix-loop-helix, residues 146 to 155, on the opposite side of the N-termini, faces the membrane in each half of the dimer. This loop contains multiple lysines that interact with phospholipids in the membrane that may act to stabilize the Nt in the cytosol. Support for this placement comes from evolving electron microscopy studies, whose images show NtAe1 to be comparable in size and shaped like a pendulum tethered to its TMD [31]. 

Moreover, the dimensions of the Nt will vary according to the intracellular pH or bicarbonate levels. Each dimer in Fig. **7** is a in a relaxed conformation at neutral pH. By relaxed, a local conformational change occurs at the N-termini that extend the axial dimensions; but also the separation between the two halves of the dimer is likely to be further apart at physiological pH than at acidic pH values. This distance is governed by the pH-dependent monomer-dimer equilibrium as dimers dissociate. Finally, the relaxed dimer in Fig. **7** allows for self-association with other dimers. Support for self-association *in-vivo* comes from evolving fluctuation fluorescence correlation spectroscopic studies that demonstrates the full-length NBCe1-A expressed in mammalian cells to be dimers with a fraction of those self-associated [32]. The self-association of NtNBCe1-A molecules prompts future experiments to investigate the molecular mechanisms of binding.

Finally Fig. **7** places a SLC4 cotransporter model, such as NtNBCe1-A, at the basolateral membrane of the cell. To the left, the Nt exists as single dimers (or less self-associated) in a closed and taut conformation at acid pHs as those observed in the study here. To the right, base (such as ammonia) enters and becomes protonated (HB), thereby neutralizing the acid load. The cotransporters now relax, permitting bicarbonate ions to bind and stabilize dimer self-associations, clustering in an open state.

## ABBREVIATIONS

NBCe1-A: = lSodium-bicarbonate cotransporter variant 1A
Nt: = N terminus
aa: = Amino acid
MALS-SEC: = Multiangle light scattering with size-exclusion chromatography
DLS: = Dynamic light scattering
